# Intermittent Fasting and Probiotics for Gut Microbiota Modulation in Type 2 Diabetes Mellitus: A Narrative Review

**DOI:** 10.3390/nu18010119

**Published:** 2025-12-30

**Authors:** Zhiwen Zhang, Shaokang Wang, Guiju Sun, Da Pan

**Affiliations:** Key Laboratory of Environmental Medicine and Engineering of Ministry of Education, Department of Nutrition and Food Hygiene, School of Public Health, Southeast University, Nanjing 210009, China; 220244290@seu.edu.cn (Z.Z.); shaokangwang@seu.edu.cn (S.W.); gjsun@seu.edu.cn (G.S.)

**Keywords:** intermittent fasting, time-restricted feeding, probiotics, gut microbiota, type 2 diabetes mellitus, *Akkermansia muciniphila*, short-chain fatty acids, glycemic control

## Abstract

**Background**: Type 2 diabetes mellitus (T2DM) is a global epidemic in which gut microbiota dysbiosis contributes to impaired glucose homeostasis and chronic inflammation. Intermittent fasting (IF) and probiotic supplementation have independently demonstrated glycemic benefits in T2DM, largely through microbiota remodeling. This narrative review synthesizes evidence up to October 2025 to clarify the microbiota-dependent mechanisms of IF and probiotics, and to evaluate the biological plausibility and preliminary clinical data for their combined application in T2DM management. **Methods**: We conducted a comprehensive literature review of preclinical and clinical studies (PubMed, Embase, Web of Science, and Cochrane Library) examining IF regimens (primarily time-restricted feeding and 5:2 protocols) and multi-strain probiotics containing Lactobacillus and Bifidobacterium species in T2DM or relevant models. Mechanistic pathways, microbial compositional shifts, and metabolic outcomes were qualitatively synthesized, with emphasis on overlapping signaling (short-chain fatty acids, bile acids, GLP-1, and barrier function). **Results**: IF consistently increases *Akkermansia muciniphila* and, variably, *Faecalibacterium prausnitzii* abundance, restores microbial circadian rhythmicity, and enhances SCFA and secondary bile acid production. Multi-strain probiotics modestly reduce HbA1c (–0.3% to –0.6%) and fasting glucose, outperforming single-strain preparations. Both interventions converge on reduced endotoxaemia and improved intestinal integrity. Preclinical models indicate potential synergy, whereas the only direct human trial to date showed neutral results. **Conclusions**: IF and probiotics engage overlapping microbiota-mediated pathways, supporting their combined use as an adjunctive strategy in T2DM. Adequately powered randomized trials incorporating deep metagenomics, metabolomics, and hard clinical endpoints are now required to confirm additive or synergistic efficacy.

## 1. Introduction

Type 2 diabetes mellitus (T2DM) affects over 500 million adults worldwide and remains a leading cause of cardiovascular disease, kidney failure, and premature mortality [1]. Despite advances in pharmacotherapy, a substantial proportion of patients fail to achieve glycemic targets, underscoring the need for effective, sustainable, and widely accessible adjunctive strategies [2]. The gut microbiota has emerged as a critical regulator of host glucose homeostasis [3,4]. Patients with T2DM typically exhibit reduced microbial diversity, depletion of butyrate-producing taxa, and impaired barrier function, collectively contributing to metabolic endotoxemia and systemic inflammation [5,6]. Interventions that favorably remodel microbial composition or function therefore represent attractive therapeutic targets.

Intermittent fasting (IF)—encompassing time-restricted feeding (TRF), alternate-day fasting, and 5:2 regimens—improves insulin sensitivity, reduces body weight, and lowers HbA1c in individuals with T2DM, often independently of calorie reduction [7,8,9]. Beyond direct metabolic effects, IF profoundly alters gut microbial ecology within days to weeks. Clinical studies, including large recent randomized trials, demonstrate that 14–18 h daily fasting windows consistently increase the relative abundance of *Akkermansia muciniphila* and, with greater inter-individual variability, *Faecalibacterium prausnitzii* [10,11,12,13,14]. These shifts coincide with restored microbial circadian rhythmicity, enhanced short-chain fatty acid (SCFA) production, and improved intestinal barrier integrity—mechanisms that likely contribute to the observed glycemic benefits [15,16,17]. Probiotic supplementation offers a complementary microbiota-directed approach. Meta-analyses of randomized controlled trials up to 2025 indicate that multi-strain preparations containing Lactobacillus and Bifidobacterium species modestly reduce fasting glucose (−0.4 to −0.9 mmol/L) and HbA1c (−0.3% to −0.6%) in T2DM, whereas single-strain interventions frequently yield null results [18,19,20,21]. The superior efficacy of multi-strain formulations is attributed to ecological complementarity, cross-feeding, and broader immunomodulatory effects [22,23,24]. Although IF and probiotics independently engage overlapping pathways—SCFA signaling, GLP-1 secretion, bile acid metabolism, and reduction in low-grade inflammation—their potential interaction remains underexplored. Preclinical models suggest that fasting-induced expansion of *Akkermansia* may create a more permissive niche for exogenous beneficial strains, whereas probiotics could stabilize IF-induced microbial shifts during refeeding phases [25,26]. However, the only dedicated human trial combining IF with a *Lactobacillus rhamnosus* strain in prediabetes reported no additive benefit [17], highlighting the need for cautious interpretation.

This narrative review, based on a structured literature search of PubMed, Embase, and Scopus and manual reference screening, aims to: (1) summarize the effects of IF and probiotic interventions on gut microbiota composition and function in T2DM; (2) delineate the microbiota-dependent mechanisms underlying their metabolic benefits, with emphasis on *Akkermansia muciniphila* and *Faecalibacterium prausnitzii*; (3) critically evaluate the biological plausibility and preliminary evidence for combined approaches; and (4) identify priority areas for future research, including adequately powered trials incorporating multi-omics and hard clinical endpoints. By clarifying the microbiota as a convergent hub for timed eating and microbial therapeutics, we seek to inform the rational design of precision nutrition strategies for T2DM.

## 2. Mechanistic Pathways Linking Intermittent Fasting, Gut Microbiota, and Type 2 Diabetess

Intermittent fasting (IF) improves glycemic control and insulin sensitivity in individuals with T2DM through multiple microbiota-dependent mechanisms. These include altered substrate availability, reinforcement of microbial and host circadian rhythms, remodeling of bile acid metabolism, increased production of short-chain fatty acids (SCFAs), and enhanced intestinal barrier function.

### 2.1. Altered Substrate Availability and Microbial Selection

Prolonged daily fasting markedly reduces the delivery of dietary polysaccharides and proteins to the distal gut, creating a low-energy, mildly acidic, mucus-reliant niche. This environment suppresses fast-growing opportunists (e.g., *Enterobacteriaceae*) while favoring mucin-degrading specialists, particularly *Akkermansia muciniphila*, and certain *Lactobacillus* species [12,13,17]. In both rodent TRF models and human Ramadan cohorts, 14–18 h daily fasting consistently increases *Akkermansia abundance* (1.5–5-fold) and often enriches *Faecalibacterium prausnitzii* [12,13,27,28,29]. These compositional shifts persist for days to weeks after fasting cessation, indicating a degree of microbial “memory” [17,30].

### 2.2. Restoration of Microbial and Host Circadian Oscillations

Feeding-fasting cycles act as a potent zeitgeber for the gut microbiome. Ad libitum feeding or mistimed meals flatten diurnal microbial oscillations, whereas TRF restores rhythmic fluctuations in *Akkermansia*, *Lactobacillus*, and *butyrate-producing* taxa [17,31,32,33,34,35,36]. In humans, early time-restricted eating (eTRE) reinstates microbial rhythmicity within 2–4 weeks. It also increases α-diversity and elevates fecal butyrate. These changes correlate with improved insulin sensitivity and reduced systemic inflammation [7,37,38,39,40]. Alignment of feeding with the host molecular clock (CLOCK/BMAL1) appears essential, as genetic disruption of Bmal1 abolishes microbial cycling and exacerbates glucose intolerance [31].

### 2.3. Bile Acid Rhythmicity and Host Signaling

**Figure 1:**
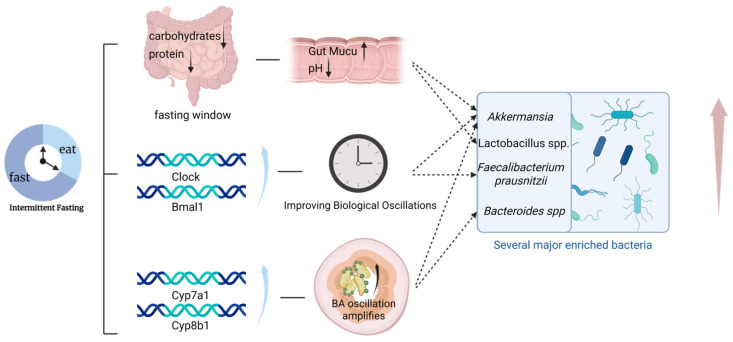
Intermittent fasting remodels gut microbiota composition in type 2 diabetes by reducing substrate availability, restoring microbial circadian rhythmicity, and amplifying bile acid oscillations, thereby selectively enriching *Akkermansia muciniphila* and other beneficial taxa to improve insulin sensitivity and glycemic control.

TRF restores diurnal oscillations in hepatic bile acid synthesis (Cyp7a1, Cyp8b1) and ileal bile acid pool size, amplifying peak-to-trough differences [41,42]. The resulting rhythmic exposure to conjugated primary bile acids favors taxa possessing bile salt hydrolase (BSH) activity, including *Akkermansia* and certain *Bacteroides* spp. Deconjugation and limited 7α-dehydroxylation alter the secondary-to-primary bile acid ratio, modulating intestinal FXR and TGR5 signaling. Although the net effect of FXR activation in T2DM remains context-dependent [43,44,45,46]. IF-induced bile acid remodeling consistently correlates with reduced hepatic glucose output and enhanced GLP-1 secretion in preclinical and small human studies [43,44,47]. These effects culminate in improved insulin signaling, as illustrated in Figure 1.

## 3. Microbiota-Mediated Mechanisms Underlying the Metabolic Benefits of Intermittent Fasting

Intermittent fasting (IF) improves glycemic control in T2DM primarily through microbiota-dependent pathways. These include enhanced short-chain fatty acid (SCFA) signaling, restored gut barrier integrity, increased GLP-1 secretion, and improved peripheral insulin sensitivity [15,16,17,48,49,50,51]. However, while human trials consistently show microbial compositional shifts and metabolic improvements, most mechanistic details are derived from preclinical models (rodent TRF studies, germ-free mouse colonization, and in vitro assays), with human evidence largely limited to correlative associations from fecal metagenomics, plasma metabolites, and indirect biomarkers [9,14,47]. Tissue-level (e.g., intestinal L-cells, liver, muscle) validation in humans remains scarce.

**Figure 2:**
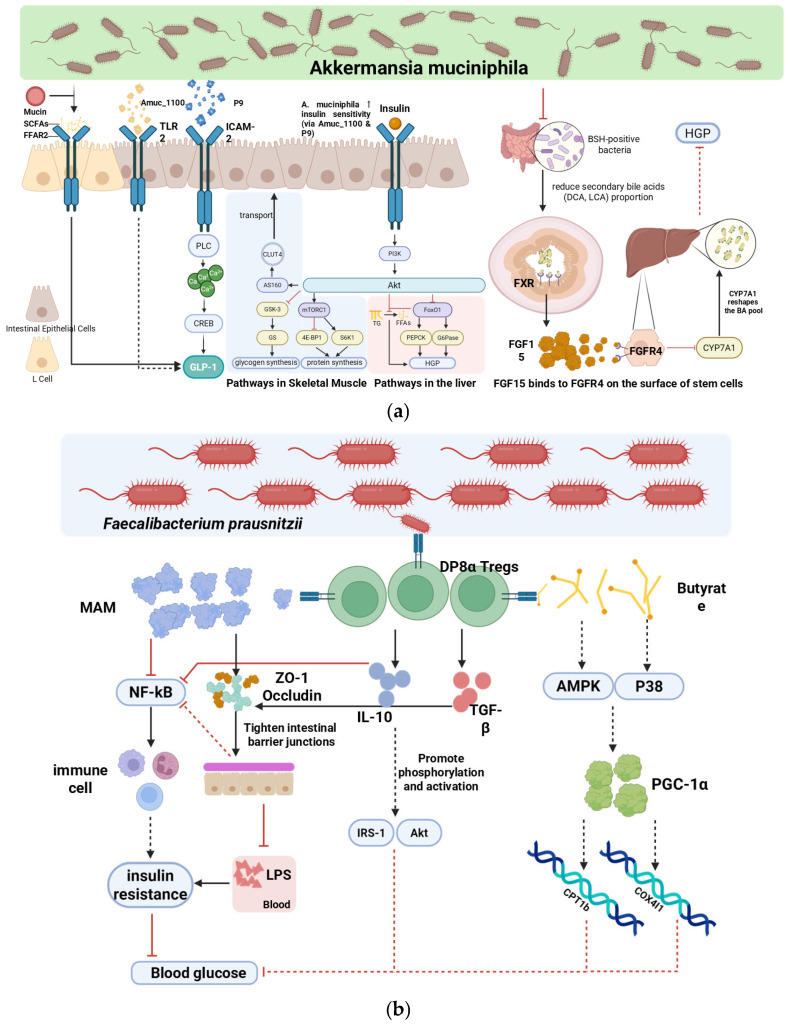
Overlapping and distinctive microbiota-mediated mechanisms by which intermittent fasting may improve glycemic control in type 2 diabetes mellitus (primarily supported by preclinical evidence). (**a**) SCFA-independent signaling pathways of *Akkermansia muciniphila*: outer-membrane protein Amuc_1100 activates TLR2, while secreted protein P9 engages the ICAM-2/PLC/Ca^2+^/CREB axis; both promote GLP-1 secretion and peripheral insulin sensitivity via PI3K–Akt. (**b**) Anti-inflammatory actions of *Faecalibacterium prausnitzii*: microbial anti-inflammatory molecule (MAM) inhibits NF-κB, and metabolites induce IL-10/TGF-β-producing CD4^+^CD8αα^+^ regulatory T cells, reducing endotoxemia and inflammation. Black arrows represent activation or promotion, while red lines denote inhibition. Solid and dashed lines indicate direct and indirect regulation, respectively. The upward and downward arrows indicate an increase and a decrease in levels, respectively.

The two most consistently modulated taxa in human IF studies are *Akkermansia muciniphila* and *Faecalibacterium prausnitzii*. Meta-analyses of trials up to 2025 confirm that 14–18 h daily fasting windows typically increase *A. muciniphila* abundance (effect size 0.6–1.2 log_2_ fold) and, less uniformly, *F. prausnitzii* [9,14,47]. Preclinical evidence proposes overlapping and distinctive contributions from these taxa, primarily through the following pathways (summarized in Figure 2).

### 3.1. Overlapping Mechanisms

In rodent models and human fecal analyses, SCFAs from both taxa are proposed to activate FFAR2/3 on L-cells. This stimulates GLP-1 and PYY secretion (acetate/propionate from *A. muciniphila*; butyrate-dominant from *F. prausnitzii*). These metabolites may also engage AMPK and PI3K–Akt signaling in peripheral tissues (liver, muscle, adipose). This suppresses gluconeogenesis (via PEPCK/G6Pase downregulation) and enhances glucose uptake via GLUT4 translocation [48,49,50,51]. Both taxa additionally appear to reinforce intestinal barrier function by upregulating tight-junction proteins (ZO-1, occludin) and mucin production, potentially reducing LPS translocation and metabolic endotoxemia—a correlation observed in small human IF cohorts [10,12,14,26,52,53].

### 3.2. Distinctive Contributions

*Akkermansia muciniphila* and *Faecalibacterium prausnitzii* are two taxa most consistently associated with IF-related metabolic effects, although mechanistic support in humans remains limited. For *A. muciniphila*, preclinical studies suggest that its outer-membrane protein Amuc_1100 (TLR2-dependent) and the secreted protein P9 (via the ICAM-2/PLC/Ca^2+^/CREB axis) may induce GLP-1 secretion largely independently of SCFAs, improving glucose homeostasis in obese and diabetic mouse models [36,47,54,55]. Human data are limited to associations between fecal *Akkermansia* levels and plasma GLP-1/insulin sensitivity [47,56].

*F. prausnitzii* is primarily implicated through anti-inflammatory mechanisms. In vitro and rodent studies demonstrate secretion of the microbial anti-inflammatory molecule (MAM), inhibition of NF-κB signaling, and induction of antigen-specific CD4^+^CD8αα^+^ regulatory T cells producing IL-10 and TGF-β, collectively reducing systemic inflammation [36,57,58,59]. Human IF trials report reduced circulating inflammatory markers correlating with *F. prausnitzii* enrichment, but causality is unproven [14,47].

In contrast, responses of *Bacteroides* spp. (including the *B. fragilis* group) to IF appear heterogeneous across studies [18,29,60]. While preclinical data suggest potential immunoregulatory effects via polysaccharide A–induced IL-10–producing Tregs through TLR2 signaling [61,62], other evidence indicates strain- and context-dependent adverse metabolic effects, including FXR dysregulation, impaired metformin efficacy, and hepatic steatosis [46]. Human metabolic implications therefore remain uncertain and warrant caution.

Collectively, these microbiota-associated mechanisms—supported predominantly by preclinical evidence—are hypothesized to converge on improved peripheral insulin sensitivity and reduced hepatic glucose output, as schematically summarized in Figure 2. Confirmation of causal relevance in humans will require longitudinal intervention studies integrating strain-resolved multi-omics and tissue-level validation.

## 4. Probiotic-Mediated Modulation of Gut Microbiota in T2DM

Meta-analyses of RCTs up to 2025 consistently demonstrate that multi-strain probiotics containing Lactobacillus and Bifidobacterium species modestly but significantly improve glycemic control in T2DM, reducing HbA1c by 0.3–0.6% and fasting glucose by 0.4–0.9 mmol/L when administered for ≥8 weeks [19,20,63]. These effect sizes are comparable to or exceed those of some oral glucose-lowering agents, with excellent tolerability. By contrast, single-strain interventions—particularly Lactobacillus monotherapy—frequently yield null or clinically negligible results [19,21,64].

Network meta-analyses confirm superior efficacy of multi-strain over single-strain formulations, attributable to ecological complementarity, cross-feeding (e.g., *Bifidobacterium* → *Faecalibacterium* butyrate production), and broader immunomodulatory coverage [20,65,66]. Subgroup analyses indicate that interventions containing ≥3 strains and total doses ≥10^9^ CFU/day are most effective [20,63].

### 4.1. Core Microbiota-Dependent Mechanisms

Multi-strain *Lactobacillus–Bifidobacterium* probiotics engage largely overlapping microbiota-mediated pathways with intermittent fasting (as detailed in Section 3), including enhanced SCFA production and FFAR2/3 signaling, reinforcement of intestinal barrier integrity, suppression of proinflammatory pathways, and restoration of peripheral insulin signaling [50,67,68,69,70]. Notably, certain aspects may be more pronounced or complementary with probiotics: Cross-feeding synergies: Bifidobacterium strains efficiently produce acetate, which promotes butyrate synthesis by indigenous producers such as *Faecalibacterium prausnitzii* [65,66]. Strain-specific immunomodulation: Selected *Lactobacillus* strains potently inhibit NF-κB activation and upregulate tight-junction proteins [68,70,71]. These complementary effects likely underlie the greater efficacy of multi-strain preparations compared to single strains.

### 4.2. Translational Limitations

Although multi-strain probiotics containing *Lactobacillus* and *Bifidobacterium* species have demonstrated modest but reproducible improvements in glycemic control in T2DM across multiple meta-analyses [19,20,63], several important limitations and sources of heterogeneity constrain their current clinical application and interpretation of efficacy. Mechanistic understanding remains predominantly derived from preclinical models and indirect circulating biomarkers. Human data providing tissue-level or causal validation of proposed pathways are sparse [68,69]. Strain-specific effects, optimal combinations, dosing thresholds, and long-term safety profiles beyond 12 months are incompletely characterized [19,20]. Furthermore, substantial inter-individual and inter-study variability in response exists. This variability is driven by differences in baseline microbiota composition, host genetics, concurrent medications (particularly metformin), dietary patterns, and degree of metabolic dysregulation. As a result, outcomes are heterogeneous, reducing the reliability of pooled estimates and limiting generalizability [3,5,12,20,70].

## 5. Intermittent Fasting Combined with Probiotics

### 5.1. Preclinical Evidence Suggesting Potential Interactions

Preclinical studies provide preliminary evidence suggesting potential interactions between intermittent fasting (IF) and multi-strain probiotics in experimental models of T2DM. In high-fat diet/STZ-induced diabetic mice, alternate-day fasting combined with a multi-strain *Lactobacillus–Bifidobacterium* cocktail resulted in greater reductions in fasting glucose, HOMA-IR, and hepatic steatosis than either intervention alone [7]. These effects were accompanied by enrichment of *Akkermansia* and *Faecalibacterium* species and increased fecal butyrate levels [7,26,48]. However, these findings are derived exclusively from animal models and cannot be directly extrapolated to human clinical efficacy.

Mechanistically, IF may favor the expansion of mucin-degrading and butyrate-producing taxa, potentially altering microbial ecology during re-feeding periods [14,23,27,34]. In theory, such shifts could influence the metabolic activity of exogenous *Lactobacillus* and *Bifidobacterium* strains, while probiotics may help stabilize fasting-induced microbial changes and modulate SCFA signaling and intestinal barrier function in metabolic disease models [50,51,65]. Importantly, these proposed interactions remain mechanistic and require validation in well-designed human studies.

### 5.2. Clinical Evidence Suggesting Potential Interactions

Clinical evidence evaluating the combined effects of IF and probiotic supplementation in T2DM remains sparse and inconclusive. To date, only one dedicated human randomized controlled trial (RCT) has explicitly examined this interaction. The PROFAST trial (2020) [17] evaluated a 5:2 IF regimen combined with a single-strain probiotic (*Lacticaseibacillus rhamnosus* HN001) versus IF plus placebo in 34 adults with prediabetes. Both groups achieved similar reductions in HbA1c (−0.4%) and body weight, with no additional glycemic benefit attributable to probiotic supplementation [17]. Interpretation of these findings is limited by the small sample size, the use of a single probiotic strain with limited evidence of efficacy in T2DM, and the inclusion of individuals with prediabetes rather than established T2DM.

A 2024 pilot RCT in medication-naïve T2DM patients (*n* = 46) investigated 16:8 time-restricted feeding combined with a multi-strain probiotic formulation. Although the combined intervention produced a numerically greater reduction in HbA1c compared with IF alone (−0.9% vs. −0.6%), this difference did not reach statistical significance (*p* = 0.07) [72]. The study was underpowered for primary glycemic endpoints due to its small sample size and the absence of stratification by baseline gut microbiota composition, which may have introduced additional variability. While secondary outcomes showed greater increases in circulating butyrate and GLP-1 levels, these findings should be interpreted as exploratory rather than indicative of clinical superiority.

Current evidence does not support clear clinical benefits of combined IF–probiotic interventions. Probiotic meta-analyses show only modest glycemic effects, and studies are heterogeneous in formulation and design [19,20,63], Well-powered RCTs are needed. These should use multi-strain or next-generation probiotics, consider baseline microbiota for stratification, and include hard endpoints such as cardiovascular outcomes or medication reduction, before additive or synergistic effects can be confirmed.

Table 1 summarizes representative preclinical and clinical studies of intermittent fasting and probiotic interventions relevant to T2DM. Several studies were conducted in non-T2DM populations or animal models and are included to illustrate mechanistic plausibility rather than direct clinical efficacy; large, long-term trials of combined interventions in T2DM remain lacking.

## 6. Discussion

IF and multi-strain probiotics independently remodel the gut microbiota in T2DM. The most consistent changes include enrichment of *Akkermansia muciniphila* and, to a lesser extent, *Faecalibacterium prausnitzii*, along with alterations in SCFA signaling, intestinal barrier function, and inflammatory tone (Section 2, Section 3 and Section 4). In human studies, these microbial shifts are generally associated with modest improvements in glycemic control. Reported HbA1c reductions range from ~0.3–0.6% for probiotic interventions and vary for IF depending on regimen, duration, and population [7,9,14,19,20,63,73,74,75]. Despite this mechanistic convergence, evidence supporting additive or synergistic clinical effects of combined IF–probiotic interventions remains limited, with only preclinical models and small pilot studies suggesting potential complementarity [7,17,72]. Table 1 provides a concise overview of representative studies and underscores the scarcity of direct human evidence for combination therapy.

Collectively, current evidence suggests several relatively consistent patterns. First, *A. muciniphila* shows the most reproducible increase across human IF trials, coinciding with restoration of microbial rhythmicity and changes in SCFA and bile acid metabolism [10,11,12,13,14,30,33,47,75]. Second, multi-strain probiotic formulations (typically ≥3 strains at doses ≥ 10^9^ CFU/day) appear more effective than single-strain preparations, plausibly through ecological complementarity, cross-feeding, and broader immunomodulatory effects [20,63,65,66]. Finally, while these observations provide a coherent mechanistic rationale for combined approaches, human evidence remains preliminary and insufficient to establish clinical superiority.

### 6.1. Limitations

Studies vary widely in design, populations, fasting protocols, and probiotic formulations, complicating synthesis and comparison [14]. Most mechanistic insights come from animal or reductionist models. Human data are mostly associative, based on correlations between fecal microbiota and circulating metabolic or inflammatory markers. Direct tissue-level or causal validation, such as FMT or strain-targeted interventions, is limited [68,69]. Key limitations therefore include the predominance of associative evidence, the scarcity of long-term trials (with most interventions lasting ≤12 weeks and limited data beyond 6 months), the lack of hard clinical endpoints (such as cardiovascular events, diabetes complications, or mortality), and the near absence of adherence and cost-effectiveness data—issues that are particularly relevant for lifestyle-based interventions such as IF.

### 6.2. Real-World Feasibility

Translation of IF–probiotic strategies into routine clinical practice faces several practical challenges. Adherence to IF may be limited by cultural and social eating patterns, lifestyle constraints (e.g., shift work, family meals), and concerns regarding hypoglycemia in medicated patients with T2DM, with dropout rates in some trials reported in the range of 20–40% [7,9,40]. Probiotic implementation is further constrained by issues of shelf stability and viability (particularly in hot or humid climates), relatively high costs for multi-strain products, variability in strain composition across commercial formulations, and uncertain engraftment in markedly dysbiotic gut ecosystems [22,66]. These considerations highlight the need for pragmatic, real-world studies that extend beyond short-term efficacy.

### 6.3. Future Directions

Future research should address the following major gaps through targeted, high-quality studies:

- Limited human evidence for combined interventions: Only one small RCT has tested IF with probiotics, showing no additive benefit [17]. Large-scale, adequately powered RCTs are needed to evaluate optimized IF regimens combined with evidence-based multi-strain probiotics.
- Short-term focus and lack of hard endpoints: Most trials are ≤12 weeks and report surrogate markers. Longer-duration studies incorporating hard clinical outcomes (e.g., cardiovascular events, diabetes complications, remission rates) are essential.
- Insufficient causal mechanistic data: Current human findings are largely correlative. Trials should integrate strain-resolved metagenomics, metabolomics, and—where ethical and feasible—tissue-level assessments to establish causality.
- Real-world translation gaps: Adherence, safety, and cost-effectiveness remain underexplored in diverse populations. Pragmatic trials in clinically representative cohorts are required to assess long-term feasibility.

These priorities will determine whether combined IF–probiotic approaches offer meaningful adjunctive benefits beyond contemporary standards of care.

In conclusion, while intermittent fasting and probiotic supplementation represent promising, microbiota-targeted adjunctive strategies for T2DM, combined approaches should currently be regarded as experimental. Rigorous, long-term evaluation against established standards of care is required before routine clinical recommendations can be justified.

## Figures and Tables

**Table 1:** Summary of evidence on intermittent fasting and probiotic interventions affecting glucose metabolism and related metabolic outcomes.

Reference	Type	Intervention	Population/Model	Sample Size	Key Microbiota Changes	Metabolic Outcomes
Liu et al. (2024) [7]	Preclinical	IF + SLBZS (prebiotic-like)	STZ-HFD diabetic mice	N = 63 (9/group)	*Akkermansiaceae*↑; *Bifidobacteriaceae*↑	FBG↓; body weight↓; OGTT AUC↓; insulin↑; dyslipidemia↓
Pavlou et al. (2023) [10]	Clinical (RCT)	8 h TRE (12:00–20:00)	Adults with T2DM and obesity	N = 75	plasma butyrate↑	HbA1c↓ (0.9% combined vs.−0.6% IF alone; *p* = 0.07); GLP-1↓
Tay et al. (2020) [17]	Clinical (RCT)	5:2 IF (600–650 kcal/day for 2 days/week) + Probiotic (*L. rhamnosus* HN001)	Adults with prediabetes	N = 26	(Microbiota composition analysis was not the primary focus of this pilot report)	HbA1c↓ (−2 mmol/mol, *p* < 0.001) and BW↓ (−5% avg.) in both groups; No additive glycemic benefit from probiotic; Mental health and social functioning in probiotic group↑ (*p* = 0.007)
Li et al. (2023) [19]	Systematic Review & Meta-analysis	Probiotic supplementation (various strains/doses)	Adults with T2DM	30 RCTs; N = 1827	Variable enrichment of beneficial taxa	FBG↓ (SMD: −0.37, *p* < 0.001); HbA1c↓ (SMD: −0.44, *p* < 0.001); Insulin↓ (SMD: −0.36, *p* = 0.004); HOMA-IR(SMD: −0.47, *p* < 0.001)↓.
Ma et al. (2025) [20]	Clinical (Network Meta)	Probiotics (multi vs. single-strain)	Adults with T2DM	30 RCTs; N = 1861	Multi-strain superior for beneficial shifts	The LAC+BIF+STR combination shows the greatest overall superiority in the cluster analysis of FPG, HbA1c, insulin, and HOMA-IR.
Li et al. (2020) [23]	Preclinical	Daily fasting (12, 16, or 20 h) for 1 month	Healthy male C57BL/6J mice	N = 60 (15/group)	*Akkermansia*↓ (only in 16 h group); *Alistipes* ↓(only in 16 h group); Changes reversible after cessation	Cumulative food intake↓ (16 & 20 h groups); No significant weight change relative to control.
Özkul et al. (2019) [29]	Clinical (Pilot)	Islamic Fasting (Ramadan); ~17 h daily fasting for 29 days	Healthy subjects	N = 9	*Akkermansia muciniphila*↓; *Bacteroides fragilis* group↓	Fasting blood glucose and total cholesterol significantly decreased, with significantly increased abundances of *Akkermansia muciniphila* and the *Bacteroides fragilis* group
Wu et al. (2025) [32]	Clinical (Observational)	Long-term fasting (10 days, water only or low calorie)	Healthy male adults	N = 13	*Bacteroidetes*↓; *Firmicutes*↓; *Akkermansia*↑; *Faecalibacterium*↓; Microbial diversity decreased initially then stabilized	Body weight↓; BM↓I; Blood glucose↓; Triglycerides; Cholesterol↓
Remely et al. (2015) [34]	Clinical (Pilot)	1-week fasting program (with laxatives) followed by 6-week probiotic intervention	Overweight/Obese adults	N = 13	*Faecalibacterium prausnitzii*↑; *Akkermansia muciniphila*↑; *Bifidobacteri*↑; *Lactobacilli*↑	Body weight↓; BM↓; Significant correlation between microbial enrichment and weight reduction.
Hejazi et al. (2024) [63]	Clinical (Meta, dose–response)	Multi-strain probiotics	Adults with T2DM	32 RCTs, N = 1920	beneficial microbial metabolites↑ (e.g., SCFAs) and improved gut barrier integrity	FBG↓; HbA1c↓; fasting insulin↓; HOMA-IR↓
Chaithanya et al. (2024) [72]	Clinical (RCT)	Multi-strain probiotic	Adults with T2DM	N = 124	No unified sequencing; strain composition product-specific	HbA1c↓; HDL-c↑; LDL-c↓; BMI↓

Abbreviations: ↑ and ↓ indicate an increase/enrichment and a decrease, respectively, following the intervention.

## Data Availability

The original contributions presented in this study are included in the article. Further inquiries can be directed to the corresponding author.

## Abbreviations

The following abbreviations are used in this manuscript:

AMP	AMP-activated protein kinase
BMAL1	Brain and muscle Arnt-like 1
BSH	Bile salt hydrolase
CREB	cAMP response element-binding protein
Cyp7a1	Cholesterol 7α-hydroxylase
eTRE	Early time-restricted eating
FFAR2/3	Free fatty acid receptor 2/3
FXR	Farnesoid X receptor
G6Pase	Glucose-6-phosphatase
GLP-1	Glucagon-like peptide-1
GLUT4	Glucose transporter type 4
HbA1c	Glycated hemoglobin A1c
HGP	Hepatic glucose production
ICAM-2	Intercellular adhesion molecule 2
IF	Intermittent fasting
IL-10	Interleukin-10
LPS	Lipopolysaccharide
MAM	Microbial anti-inflammatory molecule
NF-κB	Nuclear factor kappa B
PEPCK	Phosphoenolpyruvate carboxykinase
PI3K	Phosphatidylinositol 3-kinase
PLC	Phospholipase C
PYY	Peptide YY
RCT	Randomized controlled trial
SCFA	Short-chain fatty acid
T2DM	Type 2 diabetes mellitus
TGF-β	Transforming growth factor beta
TGR5	Takeda G protein-coupled receptor 5
TLR2	Toll-like receptor 2
TRF	Time-restricted feeding
ZO-1	Zonula occludens-1

## References

[1] Sun H., Saeedi P., Karuranga S., Pinkepank M., Ogurtsova K., Duncan B.B., Stein C., Basit A., Chan J.C.N., Mbanya J.C. (2022). IDF diabetes atlas: Global, regional and country-level diabetes prevalence estimates for 2021 and projections for 2045. Diabetes Res. Clin. Pract..

[2] Chatterjee S., Khunti K., Davies M.J. (2017). Type 2 diabetes. Lancet.

[3] Fan Y., Pedersen O. (2021). Gut microbiota in human metabolic health and disease. Nat. Rev. Microbiol..

[4] Sanders M.E., Merenstein D.J., Reid G., Gibson G.R., Rastall R.A. (2019). Probiotics and prebiotics in intestinal health and disease: From biology to the clinic. Nat. Rev. Gastroenterol. Hepatol..

[5] Qin J., Li Y., Cai Z., Li S., Zhu J., Zhang F., Liang S., Zhang W., Guan Y., Shen D. (2012). A metagenome-wide association study of gut microbiota in type 2 diabetes. Nature.

[6] Marzullo P., Renzo L.D., Pugliese G., Siena M.D., Barrea L., Muscogiuri G., Colao A., Savastano S. (2020). From obesity through gut microbiota to cardiovascular diseases: A dangerous journey. Int. J. Obes. Suppl..

[7] Liu X., Du P., Xu J., Wang W., Zhang C. (2024). Therapeutic effects of intermittent fasting combined with SLBZS and prebiotics on STZ-HFD-induced type 2 diabetic mice. Diabetes Metab. Syndr. Obes..

[8] Zhao L., Zhang F., Ding X., Wu G., Lam Y.Y., Wang X., Fu H., Xue X., Lu C., Ma J. (2018). Gut bacteria selectively promoted by dietary fibers alleviate type 2 diabetes. Science.

[9] Kootte R.S., Levin E., Salojärvi J., Smits L.P., Hartstra A.V., Udayappan S.D., Hermes G., Bouter K.E., Koopen A.M., Holst J.J. (2017). Improvement of insulin sensitivity after lean donor feces in metabolic syndrome is driven by baseline intestinal microbiota composition. Cell Metab..

[10] Pavlou V., Cienfuegos S., Lin S., Ezpeleta M., Ready K., Corapi S., Wu J., Lopez J., Gabel K., Tussing-Humphreys L. (2023). Effect of time-restricted eating on weight loss in adults with type 2 diabetes: A randomized clinical trial. JAMA Netw. Open.

[11] Han H., Xiong H., Liu Z., Liu X., Wang H., Kou J., Yi D., Shi Y., Wu H., Qiao J. (2025). Pasteurized akkermansia muciniphila Timepie001 ameliorates DSS-induced ulcerative colitis in mice by alleviating intestinal injury and modulating gut microbiota. Front. Microbiol..

[12] Derrien M., van Hylckama Vlieg J.E.T. (2015). Fate, activity, and impact of ingested bacteria within the human gut microbiota. Trends Microbiol..

[13] Hou Y., Zhai X., Wang X., Wu Y., Wang H., Qin Y., Han J., Meng Y. (2023). Research progress on the relationship between bile acid metabolism and type 2 diabetes mellitus. Diabetol. Metab. Syndr..

[14] Paukkonen I., Törrönen E.-N., Lok J., Schwab U., El-Nezami H. (2024). The impact of intermittent fasting on gut microbiota: A systematic review of human studies. Front. Nutr..

[15] Kho Z.Y., Lal S.K. (2018). The human gut microbiome—A potential controller of wellness and disease. Front. Microbiol..

[16] Cani P.D. (2019). Microbiota and metabolites in metabolic diseases. Nat. Rev. Endocrinol..

[17] Tay A., Pringle H., Penning E., Plank L.D., Murphy R. (2020). PROFAST: A randomized trial assessing the effects of intermittent fasting and *lacticaseibacillus rhamnosus* probiotic among people with prediabetes. Nutrients.

[18] Huang Y., Cao J., Zhu M., Wang Z., Jin Z., Xiong Z. (2024). Bacteroides fragilis aggravates high-fat diet-induced non-alcoholic fatty liver disease by regulating lipid metabolism and remodeling gut microbiota. Microbiol. Spectr..

[19] Li G., Feng H., Mao X.-L., Deng Y.-J., Wang X.-B., Zhang Q., Guo Y., Xiao S.-M. (2023). The effects of probiotics supplementation on glycaemic control among adults with type 2 diabetes mellitus: A systematic review and meta-analysis of randomised clinical trials. J. Transl. Med..

[20] Ma D., Zhao P., Gao J., Suo H., Guo X., Han M., Zan X., Chen C., Lyu X., Wang H. (2025). Probiotic supplementation contributes to glycemic control in adults with type 2 diabetes: A systematic review and network meta-analysis. Nutr. Res..

[21] Feizollahzadeh S., Ghiasvand R., Rezaei A., Khanahmad H., Sadeghi A., Hariri M. (2017). Effect of probiotic soy milk on serum levels of adiponectin, inflammatory mediators, lipid profile, and fasting blood glucose among patients with type II diabetes mellitus. Probiotics Antimicrob. Proteins.

[22] Moya-Pérez A., Neef A., Sanz Y. (2015). Bifidobacterium pseudocatenulatum CECT 7765 reduces obesity-associated inflammation by restoring the lymphocyte-macrophage balance and gut microbiota structure in high-fat diet-fed mice. PLoS ONE.

[23] Li L., Su Y., Li F., Wang Y., Ma Z., Li Z., Su J. (2020). The effects of daily fasting hours on shaping gut microbiota in mice. BMC Microbiol..

[24] Devaux C.A., Million M., Raoult D. (2020). The butyrogenic and lactic bacteria of the gut microbiota determine the outcome of allogenic hematopoietic cell transplant. Front. Microbiol..

[25] Mohamadshahi M., Veissi M., Haidari F., Shahbazian H., Kaydani G.-A., Mohammadi F. (2014). Effects of probiotic yogurt consumption on inflammatory biomarkers in patients with type 2 diabetes. Bioimpacts.

[26] Zhang L., Qin Q., Liu M., Zhang X., He F., Wang G. (2018). Akkermansia muciniphila can reduce the damage of gluco/lipotoxicity, oxidative stress and inflammation, and normalize intestine microbiota in streptozotocin-induced diabetic rats. Pathog. Dis..

[27] Cignarella F., Cantoni C., Ghezzi L., Salter A., Dorsett Y., Chen L., Phillips D., Weinstock G.M., Fontana L., Cross A.H. (2018). Intermittent fasting confers protection in CNS autoimmunity by altering the gut microbiota. Cell Metab..

[28] Titchenell P.M., Quinn W.J., Lu M., Chu Q., Lu W., Li C., Chen H., Monks B.R., Chen J., Rabinowitz J.D. (2016). Direct hepatocyte insulin signaling is required for lipogenesis but is dispensable for the suppression of glucose production. Cell Metab..

[29] Özkul C., Yalınay M., Karakan T. (2020). Islamic fasting leads to an increased abundance of akkermansia muciniphila and bacteroides fragilis group: A preliminary study on intermittent fasting. Turk. J. Gastroenterol..

[30] Hill C., Guarner F., Reid G., Gibson G.R., Merenstein D.J., Pot B., Morelli L., Canani R.B., Flint H.J., Salminen S. (2014). The international scientific association for probiotics and prebiotics consensus statement on the scope and appropriate use of the term probiotic. Nat. Rev. Gastroenterol. Hepatol..

[31] Liu J., Zhong Y., Luo X.M., Ma Y., Liu J., Wang H. (2021). Intermittent fasting reshapes the gut microbiota and metabolome and reduces weight gain more effectively than melatonin in mice. Front. Nutr..

[32] Wu F., Guo Y., Wang Y., Sui X., Wang H., Zhang H., Xin B., Yang C., Zhang C., Jiang S. (2025). Effects of long-term fasting on gut microbiota, serum metabolome, and their association in Male adults. Nutrients.

[33] Ozkul C., Yalinay M., Karakan T. (2020). Structural changes in gut microbiome after ramadan fasting: A pilot study. Benef. Microbes.

[34] Remely M., Hippe B., Geretschlaeger I., Stegmayer S., Hoefinger I., Haslberger A. (2015). Increased gut microbiota diversity and abundance of faecalibacterium prausnitzii and akkermansia after fasting: A pilot study. Wien. Klin. Wochenschr.

[35] Heddes M., Altaha B., Niu Y., Reitmeier S., Kleigrewe K., Haller D., Kiessling S. (2022). The intestinal clock drives the microbiome to maintain gastrointestinal homeostasis. Nat. Commun..

[36] Schmoll D., Walker K.S., Alessi D.R., Grempler R., Burchell A., Guo S., Walther R., Unterman T.G. (2000). Regulation of glucose-6-phosphatase gene expression by protein kinase bα and the forkhead transcription factor FKHR: Evidence for insulin response unit-dependent and -independent effects of insulin on promoter activity. J. Biol. Chem..

[37] Thaiss C.A., Zeevi D., Levy M., Zilberman-Schapira G., Suez J., Tengeler A.C., Abramson L., Katz M.N., Korem T., Zmora N. (2014). Transkingdom control of microbiota diurnal oscillations promotes metabolic homeostasis. Cell.

[38] Leone V., Gibbons S.M., Martinez K., Hutchison A.L., Huang E.Y., Cham C.M., Pierre J.F., Heneghan A.F., Nadimpalli A., Hubert N. (2015). Effects of diurnal variation of gut microbes and high-fat feeding on host circadian clock function and metabolism. Cell Host Microbe.

[39] Zarrinpar A., Chaix A., Yooseph S., Panda S. (2014). Diet and feeding pattern affect the diurnal dynamics of the gut microbiome. Cell Metab..

[40] Liang X., Bushman F.D., FitzGerald G.A. (2015). Rhythmicity of the intestinal microbiota is regulated by gender and the host circadian clock. Proc. Natl. Acad. Sci. USA.

[41] Ducastel S., Touche V., Trabelsi M.-S., Boulinguiez A., Butruille L., Nawrot M., Peschard S., Chávez-Talavera O., Dorchies E., Vallez E. (2020). The nuclear receptor FXR inhibits glucagon-like peptide-1 secretion in response to microbiota-derived short-chain fatty acids. Sci. Rep..

[42] Trabelsi M.-S., Daoudi M., Prawitt J., Ducastel S., Touche V., Sayin S.I., Perino A., Brighton C.A., Sebti Y., Kluza J. (2015). Farnesoid X receptor inhibits glucagon-like peptide-1 production by enteroendocrine L cells. Nat. Commun..

[43] Ferrell J.M., Chiang J.Y. (2015). Short-term circadian disruption impairs bile acid and lipid homeostasis in mice. Cell. Mol. Gastroenterol. Hepatol..

[44] Jiang C., Xie C., Lv Y., Li J., Krausz K.W., Shi J., Brocker C.N., Desai D., Amin S.G., Bisson W.H. (2015). Intestine-selective farnesoid X receptor inhibition improves obesity-related metabolic dysfunction. Nat. Commun..

[45] Chen Z., Chen H., Huang W., Guo X., Yu L., Shan J., Deng X., Liu J., Li W., Shen W. (2024). Bacteroides fragilis alleviates necrotizing enterocolitis through restoring bile acid metabolism balance using bile salt hydrolase and inhibiting FXR-NLRP3 signaling pathway. Gut Microbes.

[46] Sun L., Xie C., Wang G., Wu Y., Wu Q., Wang X., Liu J., Deng Y., Xia J., Chen B. (2018). Gut microbiota and intestinal FXR mediate the clinical benefits of metformin. Nat. Med..

[47] Yoon H.S., Cho C.H., Yun M.S., Jang S.J., You H.J., Kim J., Han D., Cha K.H., Moon S.H., Lee K. (2021). Akkermansia muciniphila secretes a glucagon-like peptide-1-inducing protein that improves glucose homeostasis and ameliorates metabolic disease in mice. Nat. Microbiol..

[48] Koh A., De Vadder F., Kovatcheva-Datchary P., Bäckhed F. (2016). From dietary fiber to host physiology: Short-chain fatty acids as key bacterial metabolites. Cell.

[49] Gasaly N., de Vos P., Hermoso M.A. (2021). Impact of bacterial metabolites on gut barrier function and host immunity: A focus on bacterial metabolism and its relevance for intestinal inflammation. Front. Immunol..

[50] Tolhurst G., Heffron H., Lam Y.S., Parker H.E., Habib A.M., Diakogiannaki E., Cameron J., Grosse J., Reimann F., Gribble F.M. (2012). Short-chain fatty acids stimulate glucagon-like peptide-1 secretion via the G-protein–coupled receptor FFAR2. Diabetes.

[51] Gao J., Mang Q., Sun Y., Xu G. (2025). Short-chain fatty acids (SCFAs) modulate the hepatic glucose and lipid metabolism of coilia nasus via the FFAR/AMPK signaling pathway In vitro. Int. J. Mol. Sci..

[52] Scherer T., O’Hare J., Diggs-Andrews K., Schweiger M., Cheng B., Lindtner C., Zielinski E., Vempati P., Su K., Dighe S. (2011). Brain insulin controls adipose tissue lipolysis and lipogenesis. Cell Metab..

[53] Effendi R.M.R.A., Anshory M., Kalim H., Dwiyana R.F., Suwarsa O., Pardo L.M., Nijsten T.E.C., Thio H.B. (2022). Akkermansia muciniphila and faecalibacterium prausnitzii in immune-related diseases. Microorganisms.

[54] Chambers E.S., Viardot A., Psichas A., Morrison D.J., Murphy K.G., Zac-Varghese S.E.K., MacDougall K., Preston T., Tedford C., Finlayson G.S. (2015). Effects of targeted delivery of propionate to the human colon on appetite regulation, body weight maintenance and adiposity in overweight adults. Gut.

[55] Plovier H., Everard A., Druart C., Depommier C., Van Hul M., Geurts L., Chilloux J., Ottman N., Duparc T., Lichtenstein L. (2017). A purified membrane protein from akkermansia muciniphila or the pasteurized bacterium improves metabolism in obese and diabetic mice. Nat. Med..

[56] Wang J., Xu W., Wang R., Cheng R., Tang Z., Zhang M. (2021). The outer membrane protein amuc_1100 of akkermansia muciniphila promotes intestinal 5-HT biosynthesis and extracellular availability through TLR2 signalling. Food Funct..

[57] Liu Q., Zhang L., Zhang W., Hao Q., Qiu W., Wen Y., Wang H., Li X. (2018). Inhibition of NF-κB reduces renal inflammation and expression of PEPCK in type 2 diabetic mice. Inflammation.

[58] Sarrabayrouse G., Bossard C., Chauvin J.-M., Jarry A., Meurette G., Quévrain E., Bridonneau C., Preisser L., Asehnoune K., Labarrière N. (2014). CD4CD8αα lymphocytes, a novel human regulatory T cell subset induced by colonic bacteria and deficient in patients with inflammatory bowel disease. PLoS Biol..

[59] Touch S., Godefroy E., Rolhion N., Danne C., Oeuvray C., Straube M., Galbert C., Brot L., Salgueiro I.A., Chadi S. (2022). Human CD4+CD8α+ tregs induced by faecalibacterium prausnitzii protect against intestinal inflammation. JCI Insight.

[60] Chen C., Liang Z., He Y., Li A., Gao Y., Pan Q., Cao J. (2023). Pravastatin promotes type 2 diabetes vascular calcification through activating intestinal bacteroides fragilis to induce macrophage M1 polarization. J. Diabetes.

[61] Round J.L., Mazmanian S.K. (2009). The gut microbiota shapes intestinal immune responses during health and disease. Nat. Rev. Immunol..

[62] Quévrain E., Maubert M.A., Michon C., Chain F., Marquant R., Tailhades J., Miquel S., Carlier L., Bermúdez-Humarán L.G., Pigneur B. (2016). Identification of an anti-inflammatory protein from faecalibacterium prausnitzii, a commensal bacterium deficient in crohn’s disease. Gut.

[63] Hejazi N., Ghalandari H., Rahmanian R., Haghpanah F., Makhtoomi M., Asadi A., Askarpour M. (2024). Effects of probiotics supplementation on glycemic profile in adults with type 2 diabetes mellitus: A grade-assessed systematic review and dose–response meta-analysis of randomized controlled trials. Clin. Nutr. ESPEN.

[64] Kocsis T., Molnár B., Németh D., Hegyi P., Szakács Z., Bálint A., Garami A., Soós A., Márta K., Solymár M. (2020). Probiotics have beneficial metabolic effects in patients with type 2 diabetes mellitus: A meta-analysis of randomized clinical trials. Sci. Rep..

[65] Rios-Covian D., Gueimonde M., Duncan S.H., Flint H.J., de los Reyes-Gavilan C.G. (2015). Enhanced butyrate formation by cross-feeding between faecalibacterium prausnitzii and bifidobacterium adolescentis. FEMS Microbiol. Lett..

[66] Kim H., Jeong Y., Kang S., You H.J., Ji G.E. (2020). Co-culture with bifidobacterium catenulatum improves the growth, gut colonization, and butyrate production of faecalibacterium prausnitzii: In vitro and In vivo studies. Microorganisms.

[67] Wang J., Tang H., Zhang C., Zhao Y., Derrien M., Rocher E., van-Hylckama Vlieg J.E.T., Strissel K., Zhao L., Obin M. (2015). Modulation of gut microbiota during probiotic-mediated attenuation of metabolic syndrome in high fat diet-fed mice. ISME J..

[68] Sabico S., Al-Mashharawi A., Al-Daghri N.M., Wani K., Amer O.E., Hussain D.S., Ahmed Ansari M.G., Masoud M.S., Alokail M.S., McTernan P.G. (2019). Effects of a 6-month multi-strain probiotics supplementation in endotoxemic, inflammatory and cardiometabolic status of T2DM patients: A randomized, double-blind, placebo-controlled trial. Clin. Nutr..

[69] Asemi Z., Zare Z., Shakeri H., Sabihi S.S., Esmaillzadeh A. (2013). Effect of multispecies probiotic supplements on metabolic profiles, hs-CRP, and oxidative stress in patients with type 2 diabetes. Ann. Nutr. Metab..

[70] Li X., Wang N., Yin B., Fang D., Jiang T., Fang S., Zhao J., Zhang H., Wang G., Chen W. (2016). Effects of lactobacillus plantarum CCFM0236 on hyperglycaemia and insulin resistance in high-fat and streptozotocin-induced type 2 diabetic mice. J. Appl. Microbiol..

[71] Singh S., Sharma R.K., Malhotra S., Pothuraju R., Shandilya U.K. (2017). Lactobacillus rhamnosus NCDC17 ameliorates type-2 diabetes by improving gut function, oxidative stress and inflammation in high-fat-diet fed and streptozotocintreated rats. Benef. Microbes.

[72] Chaithanya V., Kumar J., Vajravelu Leela K., Ram M., Thulukanam J. (2024). Impact of Multistrain Probiotic Supplementation on Glycemic Control in Type 2 Diabetes Mellitus—Randomized Controlled Trial. Life.

[73] Patterson R.E., Sears D.D. (2017). Metabolic effects of intermittent fasting. Annu. Rev. Nutr..

[74] Li G., Xie C., Lu S., Nichols R.G., Tian Y., Li L., Patel D., Ma Y., Brocker C.N., Yan T. (2017). Intermittent fasting promotes white adipose browning and decreases obesity by shaping the gut microbiota. Cell Metab..

[75] Zhou X. (2025). Glucose parameters, inflammation markers, and gut microbiota changes of gut microbiome–targeted therapies in type 2 diabetes mellitus: A systematic review and meta-analysis of randomized controlled trials. J. Clin. Endocrinol. Metab..

